# Combining evidence for association from transmission disequilibrium and case-control studies using single-nucleotide polymorphisms

**DOI:** 10.1186/1471-2156-6-S1-S106

**Published:** 2005-12-30

**Authors:** Hein Putter, Jeanine J Houwing-Duistermaat, Nico JD Nagelkerke

**Affiliations:** 1 Department of Medical Statistics and Bioinformatics, Leiden University Medical Center, University of Leiden, PO Box 9604, 2300 RC, Leiden, The Netherlands; 2 Department of Community Medicine, United Arab Emirates University, Al Ain, United Arab Emirates

## Abstract

The aim of the present analysis is to combine evidence for association from the two most commonly used designs in genetic association analysis, the case-control design and the transmission disequilibrium test (TDT) design. The cases here are affected offspring from nuclear families and are used in both the case-control and TDT designs. As a result, inference from these designs is not independent. We applied a simple logistic regression method for combining evidence for association from case-control and TDT designs to single-nucleotide polymorphism data purchased on a region on chromosome 3, replicate 1 of the Aipotu population. Combining the evidence from the case-control and TDT designs yielded a 5–10% reduction in the standard errors of the relative risk estimates. The authors did not know the results before the analyses were conducted.

## Background

To establish allelic association between single-nucleotide polymorphisms (SNPs) and a disease, broadly speaking, two types of designs dominate. The first is the classical case-control study, where the frequency of a certain allele is compared between cases and controls. The other is the transmission disequilibrium test (TDT) [1]. The TDT is a family-based method for linkage and association that is, unlike the case-control study, not sensitive to possible population stratification. The TDT and the case-control studies have essentially the same objective, namely either to identify polymorphisms (alleles) that are causally related to a phenotypic trait, or to identify polymorphisms in high linkage disequilibrium to such a causal allele. The methods only differ in methodology; the TDT looks for such alleles through associations within families whereas case-control studies do so by identifying associations within populations. For the TDT, triads consisting of parents and an affected child are needed, which may be hard to obtain. In such a situation, combining evidence for association from TDT and case-control designs may be helpful.

Such a mixture of TDT and case-control designs can occur in a number of ways. To name just a few possibilities: 1) a TDT study was originally designed, and controls were subsequently added to increase power, or linkage was found in nuclear families, and these data were combined with controls for a case-control analysis; 2) a case-control study was originally designed, and a TDT study was then set up to confirm findings, or parents of cases were later genotyped in a haplotype study in order to gain phase information [2].

Results from the separate designs are not independent, because the same cases are used in the case-control and TDT design.

In the Genetic Analysis Workshop 14, data are available on nuclear families and a modest number of controls. Ideally, one would like to combine these sources of data as efficiently as possible. In a paper by Nagelkerke et al. [3], it is shown how this can be done using simple logistic regression.

## Methods

### Statistical analysis

Consider a SNP with alleles 1 and 2. Suppose that allele 2 is the high risk allele, and that 1 is the reference allele. We assume an additive model, where the relative risks of disease of a 1/2 heterozygote and a 2/2 homozygote with respect to a 1/1 homozygote equal γ and γ^2^, respectively. The parameter γ is our parameter of interest; in what follows we refer to γ as the effect parameter and to estimates of γ as effect estimates. Let *p *be the frequency in the population of the high risk (allele 2) allele. We consider first one affected individual per nuclear family and show later how to adapt the analysis in case of multiple affected subjects. The likelihood of *p *and γ is given by

∏ *P*(genotypes of triplets | offspring affected; *p*, γ)

×

∏ *P*(genotypes of controls | *p*),

the first term corresponding to the TDT design, the second corresponding to the controls.

For a TDT family, let *G*_*o *_and *G*_*p *_denote the genotypes of offspring and parents, respectively, and let "case" denote the event that the offspring is affected. The likelihood contribution of a TDT-family is given by

*P*(*G*_*p*_, *G*_*o *_| "case") = *P*(*G*_*o *_| *G*_*p*_, "case"; *p*, γ)·*P*(*G*_*p *_| "case"; *p*, γ).

The first factor deals with transmission of alleles from parents to offspring, i.e., the TDT in its likelihood formulation [4]. The second factor essentially regains the information that was lost by using the TDT instead of the maximum likelihood estimator [3]. The complete likelihood can thus be factorized alternatively as

∏ *P*(*G*_*o *_| *G*_*p*_, offspring affected; *p*, γ)

×     (1)

∏ *P*(*G*_*p *_| offspring affected; *p*, γ)·*P*(*G*_*c *_| *p*),

where *G*_*c *_denotes the genotypes of controls. Nagelkerke et al. [3] then show that a single logistic regression with outcome *y *and two covariates *x *and *z*, given by

logit (pr(y = 1)) = exp(α + β *z *+ γ *x*)     (2)

can be carried out in order to obtain a single approximate estimate of γ from these two data sources. One covariate *z *distinguishes between whether information comes from the top line (*z *= 0) or from the bottom line (*z *= 1) of the alternative likelihood factorization (Equation 1). In the transmission part (top line), the outcome *y *equals 1 if, for a heterozygous parent, allele 2 is transmitted to the affected offspring, or 0 if allele 1 is transmitted. In case of two heterozygous parents, one transmission can be added to the dataset for each heterozygous parent. In the second part, the outcome *y *distinguishes between parent of a case (*y *= 1) or control (*y *= 0). The covariate *x *takes values 0, 0.5, and 1 for genotypes 1/1, 1/2, and 2/2, respectively (Table 1). The estimated coefficient of *x *in (Equation 1) gives an estimate of γ (effect estimate), the relative risk of having the disease with genotype 1/2 relative to 1/1 genotype. For motivation and details we refer to [3]. Note that the case-control study and the TDT can also be analyzed separately within this framework by selecting only *z *= 0 or *z *= 1 and omitting the covariate *z *(for the TDT, also the constant α has to be removed because of lack of identifiability).

For two affected offspring in a nuclear family, transmissions from the same heterozygous parent to their offspring are no longer independent, conditional on both offspring being affected. To deal with the dependencies caused by multiple affected offspring, we used the GEE (generalized estimating equations) [5] extension of logistic regression, both for the combined and for the separate case-control and TDT analyses.

### Data used

A preliminary linkage study using microsatellites showed evidence for linkage in a region on chromosome 3, in replicate 1 of the Aipotu nuclear family data in a region ranging from D03S0123 to D03S0127. Based on these findings, we purchased packages 148 through 153. All SNPs in these packages were used, again for replicate 1 of the Aipotu population. We report only on the last six SNPs from package 153, because these gave the clearest evidence for association based on the separate analyses (case-control and TDT).

As outcome we used the Kofendrerd Personality Disorder (KPD). The 100 nuclear families contained 2 (78%), 3 (16%), 4 (3%), 5 (2%), or 7 (1%) affected offspring, for a total of 233 cases. All fifty independent controls from the same data subset (replicate 1 of the Aipotu population) were also used.

The R package [6] and the geepack library was used for the GEE logistic regression analysis.

## Results

Table 2 shows the results from the case-control study, using all affected offspring from the nuclear families as cases. The standard errors are rather large because of the modest number of controls available in the case-control study.

Table 3 shows estimates (SE) from the TDT only (using logistic regression and GEE) (i.e., using the top two lines of Table 1 only), as well as from the combined analysis. Clearly, the standard errors of the estimates are reduced, on average, by about 5 to 10%. The gain in precision is reasonable, given the small number of controls used here. The other SNPs showed similar patterns (modest gains in the precision of the effect estimates in the combined analysis, compared to TDT only; results not shown).

## Discussion

The assumptions underlying our approach are essentially those that underlie either of the two constituent elements of the analysis, namely the TDT and the case-control study. In general the assumptions that underlie the case-control data, such as comparability of cases and controls and absence of population stratification, are far more stringent than those underlying the TDT. One would therefore need to verify the assumptions underlying the case-control part of the study, before the two parts can be combined. Work on testing these assumptions, notably absence of population stratifications has been published [7]. A recent paper by Epstein et al. [8] discusses a formal test of the poolability of the two designs.

It is likely that such hybrid forms of case-control and TDT designs will become more frequent in the future. The method by Nagelkerke et al. [3] is straightforward to implement, and led, in general, to increased precision of the estimate of relative risk, compared to either design separately. Standard errors of the estimates reduced by about 5 to 10%, compared to a TDT only design. With a larger number of controls, the increase in precision is likely to be larger.

Arguably the most important advantage of the present approach is that it can be implemented in any statistical package. Moreover, embedding the analysis in a generalized linear modelling framework has the benefit of diagnostic tools and the possibility of incorporating covariates into the analysis.

Our objective in this paper was very modest: to illustrate a novel method for combining evidence for association from case-control and TDT designs in a single simple analysis. The results presented in this paper are certainly promising in this particular dataset (a single replicate from a single population from the simulated Genetic Analysis Workshop 14 data). We did not determine whether the proposed method is useful or cost effective in any particular situation. Extensive simulation studies (see Epstein et al. [8] for a power comparison between combined analysis of case-control and TDT and separate analyses showing a gain of power of the combined analysis as compared to either of the separate analyses) will be necessary in order to do that.

## Conclusion

Both the case-control and the TDT analyses already showed association of SNPs B03T3056 and B03T3057 with KPD. The TDT design yielded considerably smaller standard errors than the case-control design. Combining the evidence from the case-control and TDT studies yielded a further 5–10% reduction in the standard errors of the effect estimates, compared to the TDT-only design.

## Abbreviations

GEE: Generalized estimating equations

KPD: Kofendrerd Personality Disorder

SNP: Single-nucleotide polymorphism

TDT: Transmission disequilibrium test

## Authors' contributions

HP performed the analyses and wrote the manuscript. All authors participated in the development of the methods and in the interpretation of the results of the analysis. All authors read and approved the final manuscript.

## Figures and Tables

**Table 1:** Summary of data preparation for the logistic regression of equation (1) with outcome *y *and covariates *x *and *z*

*y*	*x*	*z*	Comments
1	1	0	TDT, heterozygous parent, allele 2 transmitted
0	1	0	TDT, heterozygous parent, allele 1 transmitted
1	*i*/2	1	Parent of case, *i *copies of allele 2
0	*i*/2	1	Control, *i *copies of allele 2

**Table 2:** Results from the case-control analysis

SNP	γ	SE	*z*	*P*
B03T3055	0.345	0.449	0.768	0.44
B03T3056	-2.900	0.573	-5.061	4.20 × 10^-7^
B03T3057	-1.994	0.590	-3.380	7.30 × 10^-4^
B03T3058	-0.233	0.479	-0.486	0.13
C03R0281	-0.146	0.446	-0.327	0.74
B03T3060	-0.699	0.688	-1.016	0.31

**Table 3:** Results from TDT only and from the combined analysis

	TDT				Combined analysis			
		
SNP	γ	SE	*z*	*P*	γ	SE	*z*	*p*
B03T3055	-0.315	0.169	-1.858	0.063	-0.209	0.153	-1.368	0.17
B03T3056	-1.114	0.192	-5.797	6.70× 10^-9^	-1.245	0.189	-6.597	4.20 × 10^-11^
B03T3057	-0.535	0.163	-3.288	0.001	-0.62	0.156	-3.962	7.40 × 10^-5^
B03T3058	-0.571	0.193	-2.952	0.0032	-0.467	0.162	-2.89	0.0039
C03R0281	-0.355	0.174	-2.047	0.041	-0.284	0.157	-1.806	0.071
B03T3060	-0.199	0.206	-0.967	0.33	-0.229	0.192	-1.194	0.23
